# Correlation between* Taijin-Kyofu-sho* and Attention Deficit Hyperactivity Disorder among University Students: A Self-Reported Assessment Study

**DOI:** 10.1155/2019/7953123

**Published:** 2019-03-04

**Authors:** Kosuke Kajitani, Rikako Tsuchimoto, Tomoko Matsushita, Hideaki Fukumori

**Affiliations:** Center for Health Sciences and Counseling, Kyushu University, 744, Motooka, Nishi-ku, Fukuoka 819-0395, Japan

## Abstract

*Taijin-Kyofu-sho* (TK) is regarded as a culture-bound anxiety disorder in East Asian counties. Despite its earlier discovery in Japan, fewer studies have focused on TK than on social anxiety disorder (SAD) and even fewer on TK comorbidity with developmental disorders. Thus, we examined the association between TK and attention deficit hyperactivity disorder (ADHD) among Japanese university students. A total of 673 students (500 male, 173 female) were assessed on the Japanese version of Liebowitz Social Anxiety Scale (LSAS-J), TK scale, and adult ADHD Self-Report Scale (ASRS; version 1.1). On the TK scale and LSAS-J, 17.4 and 10.3 percent of students, respectively, exceeded the cut-off value. Furthermore, ASRS scores more strongly correlated with TK scale than LSAS-J scores (TK scale:* r* = 0.427; LSAS-J:* r* = 0.330). To evaluate how TK or SAD with ADHD affects those scores, we divided subjects into four groups: healthy subjects, subjects with TK, those with SAD, and those with both disorders. The total ASRS score was significantly higher in TK-only subjects than in healthy subjects (*p* < 0.0001). However, there was no significant difference between scores of healthy and SAD-only subjects (*p* = 0.281). Our results indicate a possible link between ADHD and later development of TK in Japan.

## 1. Introduction

Social anxiety disorder (SAD), also called social phobia, is an anxiety disorder characterized by a marked fear or anxiety of social situations, including meeting people, talking in meetings or groups, starting conversations, eating while being observed, or being seen or speaking in public [1]. A person with SAD is afraid that he or she will make mistakes, look bad, or be embarrassed or humiliated in front of others. The onset of SAD occurs in childhood or adolescence by 11 years in about 50% and by 20 years in 80% of individuals [2]. SAD can negatively interfere with communication skills, general well-being, social life, and academic performance. Although several studies have shown that SAD is one of the most prevalent psychiatric disorders [3], SAD is considered a relatively new anxiety disorder according to classifications of the Diagnostic and Statistical Manual of Mental Disorders (DSM). Although social phobia (*phobies sociales*), the name by which SAD was formerly referred, was first coined by Janet in his monograph published in the early 1900s [4], there was no corresponding concept of SAD in the US until Marks and Gelder divided phobic disorders into three types: agoraphobia, specific phobia, and social phobia [5]. For this reason, SAD was regarded as “a neglected anxiety disorder” [6].

Meanwhile, SAD in Japan has been described since the 1920s. Morita initially described the features of SAD as “*Taijin-Kyofu-sho *(TK)” literally meaning the disorder (*sho*) of fear (*kyofu*) of interpersonal relations (*taijin*) [7, 8]. TK, a form of SAD, is regarded as a culture-bound anxiety disorder in Japan and other East Asian counties [9]. The fifth edition of the DSM (DSM-5) describes the characteristic features of TK as persistent and excessive fears of giving offense to others in social situations by their physical characteristics, such as blushing, gaze, or one's body odor [10]. Several discussions have been published concerning the concept of TK. For example, some groups in East Asia classify TK into two subtypes [11, 12]; the tension or general type and the offensive type. In the tension type, patients fear being looked down upon because of a physical manifestation of anxiety or embarrassment, such as blushing, in social situations. They also feel shame for experiencing these anxieties and fears and therefore, avoid social situations where the anxiety might be provoked. These features resemble SAD regarding “fear of being noticed” [11]. In contrast, the offensive type, which is regarded as the severe type of TK in Japan, is characterized by the fear of offending or disgusting others by eye contact or body odor. Under the DSM diagnostic system, this type is classified as obsessive-compulsive disorder, body dysmorphic disorder, delusional disorder somatic type, or paranoid or schizotypal personality disorders, as observed in western countries [13]. Thus, although TK has been regarded as a type of SAD, it is still controversial whether these two disorders are identical because of several differences in their clinical features.

Although the concept of SAD is relatively new compared to TK, a growing body of research has revealed important findings on the etiology, diagnosis, cause, and treatment of this disorder. For example, several studies have reported the relationship between SAD and other psychiatric disorders, including mood disorder, obsessive compulsive disorder, panic disorder, autism, and attention deficit and hyperactivity disorder (ADHD) [2, 14–17]. From the viewpoint of developmental disorders, the assessment of SAD comorbidity is especially important in clinical practice, because the average age of SAD onset is during teenage life. This suggests that developmental disorders may trigger or exacerbate SAD symptoms during early developmental stages. Indeed, several studies have indicated the high prevalence of SAD in children with ADHD [18, 19] and the high rate of ADHD history in adult patients with SAD [20–22].

Despite the earlier discovery of TK in Japan, fewer studies have focused on TK than on SAD, and much fewer on its comorbidity with developmental disorders. To the best of our knowledge, there is no study on the relationship between TK and ADHD. Furthermore, there are limited studies regarding TK on university students [7, 10, 23], although the typical age of TK onset is adolescence and early adulthood [4]. The aims of this study were to estimate the frequency of TK occurrence in Japanese university students and examine the association between TK and ADHD using self-reported screening tests.

## 2. Materials and Methods

### 2.1. Study Design and Subjects

This was an institution-based cross-sectional study conducted in Kyushu University from April 2015 to October 2017. All participants were Japanese students who volunteered to participate in the study and were recruited through the department of Interdisciplinary Graduate School of Engineering Sciences and Faculty of Arts and Science in Kyushu University. In the class of psychology or safety and health education, we explained the study outline and asked for volunteers. We excluded subjects who had previously completed the same psychological tests in the authors' class to avoid duplicate subjects. In total, 673 students (males: 500, females: 173) completed all psychological tests. The mean age of the participants was 21.1 years (SD = 2.12 years). This study was approved by the Ethics Committee of the Faculty of Arts and Science, Kyushu University, Japan.

### 2.2. Measures

The TK scale, developed by Kleinknecht et al. (1997), consists of 31 items associated with TK symptoms. These items include symptoms that most highly differentiate patients with TK from nonpatients in Japan [7]. The subjects were instructed to rate items on a seven-point scale (1 = totally false to 7 = exactly true). Sixteen items (item numbers 1, 5, 6, 10, 11, 15, 17, 18, 22, 23, 24, 25, 26, 27, 28, and 30) involved symptoms of the offensive TK type (e.g., “I'm afraid that my presence will offend others”). The other 15 items (item numbers 2, 3, 4, 7, 8, 9, 12, 13, 14, 16, 19, 20, 21, 29, and 31) reflected features of the tension TK type (e.g., “I cannot feel relaxed when I chat with my friends”) [12]. For the analysis, we used the sum of scores in offensive type-related items (o-TK: ranging from 16 to 112), the sum of scores in tension type-related items (t-TK: ranging from 15 to 105), and the total TK-scale scores in all 31 items (ranging from 31 to 217). According to a previous report, we defined as “TK cases” subjects who scored higher than one standard deviation above the mean score on the TK scale [7].

We used the Japanese version of the Liebowitz Social Anxiety Scale (LSAS-J) for the present study [24]. LSAS-J consists of 24 items that assess the extent of social interactions and performance in situations relevant to SAD symptoms [6]. Subjects were asked to rate the 24 items on a 4-point scale (0 =* none* to 3 =* severe*) based on the fear felt during specific situations (Fear or Anxiety section), and then to rate them again based on the avoidance of a situation (Avoidance section). Total fear/anxiety and total avoidance scores were both 0 to 72, and thus, the total LSAS-J score ranged from 0 to 144. Previous reports have determined the cut-off value (COV) of the LSAS-J for SAD (total LSAS-J score: 60) [25–28]. Therefore, we classified the study subjects according to their total LSAS-J score as follows; SAD group (60 or higher) and non-SAD group (less than 60).

Adult ADHD Self-Report Scale version 1.1 (ASRS-v1.1) is a self-reported questionnaire designed to screen for adult ADHD [29], which consists of 18 items rated on a 5-point scale (0 = never, 1 = rarely, 2 = sometimes, 3 = often, and 4 = very often). We calculated the total score by summing the values of all items (ranging from 0 to 72). The higher the score, the more pronounce the symptoms. Furthermore, we calculated two subscales: inattention (IA) and hyperactivity/impulsivity (H/I), according to previous reports [30, 31]. The 18 items were divided into two groups as follows: items related to IA (item numbers 1, 2, 3, 4, 7, 8, 9, 10, and 11) and those related to H/I (item numbers 5, 6, 12, 13, 14, 15, 16, 17, and 18). Thus, the total score of IA or H/I was between 0 and 36.

### 2.3. Data Analysis

**Figure 1:**
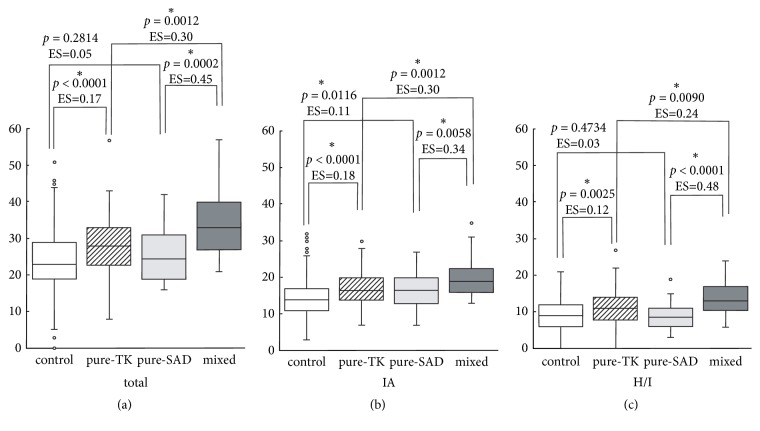
Box-and-whisker plots of adult ASRS scores. (a) Comparison of the total score on the ASRS. (b) Comparison of scores for IA- (inattention-) related items of the ASRS. (c) Comparison of scores for H/I- (hyperactivity/impulsivity-) related items of the ASRS. White boxes: healthy control group [LSAS-J: cut-off value (COV) < 60/TK: COV < 122]. Boxes with oblique lines: pure-TK group (LSAS-J: COV < 60/TK: COV ≥ 122). Light gray boxes: pure-SAD group (LSAS-J: COV ≥ 60/TK: COV < 122). Dark gray boxes: mixed group (LSAS-J: COV ≥ 60/TK: COV ≥ 122). Differences among all groups were statistically significant (p < 0.001, Kruskal-Wallis test). Comparison between two groups was performed using the Mann-Whitney* U* test with Bonferroni correction (^*∗*^p < 0.0125). ES: effect size; SAD: social anxiety disorder; ASRS: ADHD Self-Report Scale; LSAS-J: the Japanese version of the Liebowitz Social Anxiety Scale; TK:* Taijin-Kyofu-sho*.

Data were analyzed using SPSS version 23.0 (IBM Corporation, Armonk, NY, USA). The Shapiro-Wilk test was employed to evaluate whether each set of measures was a normally distributed trait. Comparisons between males and females were performed using Mann-Whitney* U* test. Data are shown as median (interquartile range: IQR) in Table 1 and Figure 1.

The relationships between pairs of variables were examined using Spearman's correlation coefficient (Table 2). Statistical significance was set at* p *< 0.05, except for the analysis in Figure 1. In Figure 1, we compared the differences between the four groups, using the Kruskal-Wallis test. After Bonferroni correction, the statistical significance was defined as p < 0.0125 for multiple comparisons, using Mann-Whitney* U* test.

## 3. Results

### 3.1. LSAS-J, TK-Scale, and ASRS Scores

Table 1 shows the median and IQR for all questionnaires used in the present study. The median total score on the LSAS-J was 30.0 (IQR: 18.0-43.0), the median score for items of the LSAS-J related to fear was 19.0 (IQR: 12.0-27.0), and for items related to avoidance 11.0 (IQR: 5.0-18.5). Cronbach's alpha of the LSAS-J was 0.95. For each group of LSAS-J items, the scores of females were significantly higher than those of males [male* vs* female median (IQR), total: 28.0 (16.0-54.0)* vs* 38.0 (23.0-53.5), ^*∗*^*p* < 0.01; fear: 18.0 (11.0-25.0)* vs* 24.0 (15.0-31.0), ^*∗*^*p* < 0.01; avoidance: 10.0 (4.0-17.0)* vs* 14.0 (7.0-23.5), ^*∗*^*p* < 0.01].

The median total score on the TK scale was 88.0 (IQR: 61.0-113.0), the median score for o-TK was 42.0 (IQR: 31.0-56.5), and for t-TK was 44.0 (IQR: 30.0-56.0). Cronbach's alpha was 0.96. Similarly to LSAS-J, in each group of TK-scale items, the scores of females were significantly higher than those of males [male* vs* female median (IQR), total: 82.0 (57.3-109.0)* vs* 100.0 (72.0-120.5), ^*∗*^*p* < 0.01; o-TKS: 41.0 (29.0-55.0)* vs* 49.0 (35.0-61.5), ^*∗*^*p* < 0.01; t-TKS: 42.0 (28.3-54.0)* vs* 50.0 (37.0-60.0), ^*∗*^*p* < 0.01]. According to Kleinknecht et al., we defined as “TK cases” subjects who scored higher than one standard deviation above the mean score on the TK scale [7]. The mean total score and standard deviation were 88.4 and 33.1, respectively. Finally, we defined the COV of the TK scale as 122.

The median total score on the ASRS was 24.9 (IQR: 19.0-30.0), the median score for IA-related items was 15.0 (12.0-18.0), while the one for H/I-related items was 10.0 (IQR: 7.0-13.0). Cronbach's alpha was 0.84. There were no significant differences between males and females in this case for any item group [male* vs* female median (IQR), total: 25.0 (19.0-30.0)* vs* 24.0 (20.0-30.0),* p* = 0.65; IA: 15.0 (12.0-18.0)* vs* 15.0 (12.0-18.0),* p* = 0.36; H/I: 10.0 (7.0-13.0)* vs* 9.0 (6.5-12.0),* p* = 0.12]

### 3.2. Correlations among Measures

Next, we examined the correlation among the different measures obtained in this study. The results are shown in Table 2. We found strong and significant correlations between scores on the LSAS-J and TK scale (LSAS-J: fear* vs* avoidance,* r* = 0.675; TK scale: o-TK* vs* t-TK,* r* = 0.893). However, although statistically significant, the inter-correlation of ASRS scores was moderate (ASRS: IA* vs* H/I,* r* = 0.487). The correlation between LSAS-J and TK-scale scores was also strong and significant, especially between scores in fear-related items in the LSAS-J and in t-TK items (*r* = 0.620). Additionally, there were significant but weak correlations between subscales of the ASRS and LSAS-J (H/I* vs* fear,* r* = 0.222; H/I* vs* avoidance,* r* = 0.181). There was also a moderate yet significant correlation between subscales of the TK scale and ASRS (IA* vs* o-TK,* r* = 0.420; IA* vs* t-TK,* r* = 0.415).

### 3.3. Correlation of TK Features with ADHD Severity

Next, to examine the correlation of TK features and the severity of ADHD, we compared the score on the ASRS (total, IA, and H/I) between subjects with high and low scores on the TK-scale. Tarumi et al. investigated subjects with SAD at sub-clinical level, using the TK scale and LSAS-J, and found a group of individuals with symptomatic profiles that fit the o-TK [23]. Interestingly, this group had high TK-scale scores despite having relatively low LSAS-J scores [23]. Taking these findings into consideration, we divided our subjects into four groups (Table 3); healthy controls [LSAS-J (COV < 60)/TK(COV < 122)], subjects with TK [pure-TK; LSAS-J (COV < 60)/TK(COV ≥ 122)], subjects with SAD [pure-SAD; LSAS-J (COV ≥ 60)/TK(COV < 122)], and subjects with both TK and SAD [mixed; LSAS-J (COV ≥ 60)/TK(COV ≥ 122)]. In total, 69 (10.3%) subjects exceeded the COV (≥ 60) on the LSAS-J, while 117 (17.4%) subjects exceeded the COV (≥122) on the TK scale. There was a significant difference among the four groups in each ASRS score (Kruskal-Wallis test,* p* < 0.001).

As shown in Figure 1(a), the total score on the ASRS was significantly higher in pure-TK subjects than in healthy subjects [*p* < 0.0001, effect size (ES) = 0.17]. Furthermore, this score was significantly higher for subjects with both disorders than for SAD-only (pure-SAD) subjects (*p* = 0.0002, ES = 0.45). However, there was no significant difference between healthy and pure-SAD subjects (*p* = 0.2814, ES = 0.05). With respect to the comparison between pure-TK and pure-SAD, we found no statistically significant difference (*p* = 0.2814, ES = 0.0131). There was also a statistically significant difference between pure-TK and mixed subjects (*p* = 0.0012), although the effect size was moderate (ES = 0.30). Regarding the comparison between healthy subjects and mixed subjects, a statistically significant difference was observed (*p* < 0.0001, ES = 0.27). As shown in Figure 1(b), the score for IA-related items of the ASRS was slightly, but significantly, higher in pure-TK subjects than in healthy subjects (*p* < 0.0001, ES = 0.18). This score was moderate but significantly higher in the mixed subjects than in pure-SAD subjects (*p* = 0.0058, ES = 0.34), as well as in pure-TK subjects (*p* = 0.0012, ES = 0.30). There was a statistically significant difference between healthy and pure-SAD subjects, although the effect size was small (*p* = 0.0116, ES = 0.11). Though the difference between pure-TK and pure-SAD was nonsignificant (*p* = 0.8205, ES = 0.02), we found a statistically significant difference between healthy subjects and mixed subjects (*p* < 0.0001, ES = 0.28). As shown in Figure 1(c), the score for H/I-related items of the ASRS was slightly, but significantly, higher in pure-TK subjects than in healthy subjects (*p* = 0.0025, ES = 0.12). This score was moderately and significantly higher in mixed subjects than in pure-SAD subjects (*p* < 0.0001, ES = 0.48). Although there was no statistically significant difference between healthy and pure-SAD subjects (*p* = 0.4734, ES = 0.03), there was a difference between pure-TK and mixed subjects (*p* = 0.0090, ES = 0.24). There was no statistically significant difference between pure-TK and pure-SAD (*p* = 0.017, ES = 0.23); however, the difference between healthy subjects and mixed subjects was statistically significant (*p* < 0.0001, ES = 0.21).

## 4. Discussion

In the current study, we found that 17.4% and 10.3% of university students exceed the COV on the TK scale and the LSAS-J, respectively. Moreover, we discovered that female university students score higher on both the TK scale and LSAS-J than male students. Furthermore, we demonstrated that ASRS scores strongly correlate with TK scale rather than LSAS-J scores.

ADHD, characterized by chronic attention and impulse control deficits, was historically regarded as a disorder of childhood. However, prospective studies have shown that ADHD often persists into adulthood [32, 33], while a previous meta-analysis indicated that the prevalence of adult ADHD is 2.5-4.9% [34, 35]. Furthermore, comorbidity studies have demonstrated that 47% of adults with ADHD have anxiety disorders, including SAD (29%) [14]. Thus, there is an accumulation of research reporting the relationship between ADHD and SAD. However, to the best of our knowledge, this is the first report showing the correlation of ADHD and TK, which is a culture-bound form of SAD.

There are several studies on SAD using the LSAS-J for university students, and previous reports have shown that the total score on this scale ranges between 30 and 45 [10, 36–39]. In agreement, the median score on the LSAS-J in our study was 30.0. Epidemiological studies have consistently shown a greater proportion of females meeting the criteria for SAD [40–42]. Similarly, we also found that females scored significantly higher than males on this scale. Thus, our results on the LSAS-J are in line with previous reports.

Although there are several reports on the etiology of TK, very few have focused on young adults. Kleinknecht et al. developed the TK scale and compared the scores of U.S. and Japanese university students [7]. The authors found that Japanese students had higher scores than U.S. students (90.53 ± 29.04* vs* 80.86 ± 26.88). Essau et al. investigated SAD and TK both in Japan and England, using the TK scale, and showed that the mean score on this scale was higher in Japanese students than in English students (93.45 ± 37.8* vs* 72.51 ± 32.1) [43]. In our study, the median score (IQR) for both male and female students was 88.0 (61.0-113.0), similar to scores observed in previous studies. We also showed a statistically significant difference in TK-scale scores between male and female. In agreement, two studies by Essau et al. also showed that females scored significantly higher on the TK scale than males [43, 44].

ASRS is widely used as a diagnostic tool for the assessment of ADHD, and many studies have included young adults, as well as university students. For example, Takeda et al. examined the reliability and validity of the Japanese version of the ASRS for different subjects, including 894 university students, and showed that their mean score on this scale was 25.3 [45]. Yeh et al. reported a mean score of 24.8 for college students [46]. Consistently, we found a median score of 24.0 on the ASRS.

We observed a moderate correlation between the total ASRS and TK-scale scores and a weak correlation between the total ASRS and LSAS-J scores. The ASRS subscale IA showed moderate correlation with both TK subscales (o-TK and t-TK) but a weak correlation with LSAS-J subscales (fear and avoidance). In addition, H/I weakly correlated with TK subscales and even more weakly with LSAS-J subscales. Koyuncu et al. reported a high frequency of childhood inattentive type ADHD in patients with SAD in Turkey [16] and that impulsiveness and SAD symptom severity are interrelated [20]. Thus, specific symptoms of ADHD show strong connection with SAD symptoms and severity; however, our results indicate that patients with ADHD in Japan tend to exhibit TK rather than SAD. We found that pure-TK subjects have higher scores on the ASRS (total,* IA* and H/I) than healthy controls, whereas there were no statistically significant differences between pure-SAD and healthy subjects. These results indicate that students with clinical features of TK rather than SAD are more likely to present clinical features of ADHD. Subjects in the mixed group, with both SAD and TK features, showed higher ASRS scores than the other groups, suggesting two possible explanation: (1) the features of TK and SAD may independently correlate with the clinical symptoms of ADHD and (2) the mixed group may have included subjects whose severe psychopathology correlated with the symptoms of ADHD. Either way, future research should further characterize the relationship between subjects in the mixed group and ADHD symptoms.

Cultural undercurrents in Japan may contribute to the positive correlation between the scores on the ASRS and TK-scale. In Western countries, including the US, society is characterized by individualism; one receives praise for standing out from the crowd and being unique [7, 47]. In contrast, East Asian societies, including Japan, are characterized by collectivism [48], where people need to sensitively perceive the feelings of others because “not bothering others” and “cooperativeness” are regarded as virtues; i.e., the fear of being disliked and even ostracized plays a prominent role in collectivistic societies like Japan [49]. Thus, the cultural background in East Asia, which easily promotes the development of personalities that are directed by others [50], may produce the feature of TK, i.e., “excessive fear of giving offense to others in social situations”.

Koyuncu et al. reported that higher rates of emotional traumatic experiences in patients with SAD and childhood ADHD than in those without ADHD [20]. This suggests that emotional trauma may constitute a link between ADHD and later development of SAD. Indeed, a number of studies have found that children with ADHD are subject to peer rejection or peer victimization [51–53] and are likely to experience great stress and trauma in interpersonal situations [54–56]. In summary, children with features of ADHD in East Asia acquire the habit of reading too much into other people's feelings, so as not to repeat the conflict with others, and this habit may transform into the features of TK.

### 4.1. Strength and Limitations of This Study

This is the first study to examine the association between TK and ADHD. However, the present study has some limitations. First, all participants were recruited from a single University. In Kyushu University, about 70% of the students are male, suggesting that our results cannot be generalized to all young adults in Japan. Moreover, in order to show that our results are specific to East Asia, it is important to compare the impact of ADHD on TK between students in Western countries and students in East Asia. Second, we relied on self-reported assessment to diagnose SAD and TK. In general, a clinical diagnosis is determined by a psychiatric interview, according to operational diagnostic criteria. Therefore, a clinical evaluation by structured interview would be required for the accurate diagnosis of TK and ADHD. Finally, we did not investigate the psychological background of each subject, such as self-construal, parental relation, and experience of traumatic events, which may be related to the pathogenesis of TK and ADHD. This information will deepen the understanding of the relationship between TK and ADHD.

## 5. Conclusions

The current study provided important information on the frequency of TK symptoms among young adults. Furthermore, we showed that females exhibit significantly higher scores than males on both the LSAS-J and TK-scale. In addition, we found that ASRS scores strongly correlate with TK-scale scores, suggesting a possible link between ADHD and later development of TK. Further studies are needed to elucidate the impact of ADHD on TK from the viewpoint of cross-cultural comparison, self-construal, and traumatic events during early developmental stages. In particular, as the two distinct features of TK (o-TK and t-TK) complicate the understanding of its psychopathology, the development of a screening method for o-TK and/or t-TK will help to determine the relevance of TK to other psychiatric disorders.

## Figures and Tables

**Table 1:** Scores on the LSAS-J, TK scale, and ASRS.

measures	T median (IQR)	M median (IQR)	F median (IQR)	*p *value (M *vs* F)
*LSAS-J*				
total	30.0 (18.0-43.0)	28.0 (16.0-54.0)	38.0 (23.0-53.5)	0.00^*∗*^
fear	19.0 (12.0-27.0)	18.0 (11.0-25.0)	24.0 (15.0-31.0)	0.00^*∗*^
avoidance	11.0 (5.0-18.5)	10.0 (4.0-17.0)	14.0 (7.0-23.5)	0.00^*∗*^

*TK-scale*				
total	88.0 (61.0-113.0)	82.0 (57.3-109.0)	100.0 (72.0-120.5)	0.00^*∗*^
o-TKS	42.0 (31.0-56.5)	41.0 (29.0-55.0)	49.0 (35.0-61.5)	0.00^*∗*^
t-TKS	44.0 (30.0-56.0)	42.0 (28.3-54.0)	50.0 (37.0-60.0)	0.00^*∗*^

*ASRS*				
total	24.0 (19.0-30.0)	25.0 (19.0-30.0)	24.0 (20.0-30.0)	0.65
IA	15.0 (12.0-18.0)	15.0 (12.0-18.0)	15.0 (12.0-18.0)	0.36
H/I	10.0 (7.0-13.0)	10.0 (7.0-13.0)	9.0 (6.5-12.0)	0.12

T: total; M, male; F: female; LSAS-J: the Japanese version of the Liebowitz Social Anxiety Scale; TK scale: *Taijin-Kyofu-sho* scale; o-TK: offensive type TK; t-TK: tension type TK; ASRS: adult ADHD Self-Report Scale; IA: inattention; H/I: hyperactivity/impulsivity; IQR: interquartile range. ^*∗*^*p* < 0.01:, Mann-Whitney *U*-test.

**Table 2:** Correlations among measures.

		LSAS-J			TK scale			ASRS	
	total	fear	avoidance	total	o-TK	t-TK	total	IA	H/I
LSAS-J									
total	--								
fear	.919^*∗∗*^	--							
avoidance	.899^*∗∗*^	.675^*∗∗*^	--						
TK scale									
total	.584^*∗∗*^	.601^*∗∗*^	.472^*∗∗*^	--					
o-TK	.536^*∗∗*^	.552^*∗∗*^	.435^*∗∗*^	.972^*∗∗*^	--				
t-TK	.601^*∗∗*^	.620^*∗∗*^	.484^*∗∗*^	.972^*∗∗*^	.893^*∗∗*^	--			
ASRS									
total	.330^*∗∗*^	.341^*∗∗*^	.272^*∗∗*^	.427^*∗∗*^	.430^*∗∗*^	.403^*∗∗*^	--		
IA	.356^*∗∗*^	.367^*∗∗*^	.292^*∗∗*^	.427^*∗∗*^	.420^*∗∗*^	.415^*∗∗*^	.868^*∗∗*^	--	
H/I	.214^*∗∗*^	.222^*∗∗*^	.181^*∗∗*^	.318^*∗∗*^	.331^*∗∗*^	.288^*∗∗*^	.839^*∗∗*^	.487^*∗∗*^	--

^*∗∗*^Correlation is significant at < 0.001. LSAS-J: the Japanese version of the Liebowitz Social Anxiety Scale; TK: *Taijin-Kyofu-sho*; o-TK: offensive type TK; t-TK: tension type TK; ASRS: adult ADHD Self-Report Scale; IA: inattention; H/I: hyperactivity/impulsivity.

**Table 3:** Cut-off values and number of subjects in each group.

	*TK scale COV (≥ 122)*		*Total*
	Not exceeded: TK(−)	Exceeded: TK(+)	
*LSAS-J COV (*≥* 60)*			
Not Exceeded: SAD(−)	healthy controls	pure-TK	
	526 (78.2)	78(11.6)	604 (89.8)
Exceeded: SAD(+)	pure-SAD	mixed-type	
	30(4.4)	39(5.8)	69 (10.2)

*Total*	556(82.6)	117(17.4)	673(100)

COV: cut-off value (%); LSAS-J: the Japanese version of the Liebowitz Social Anxiety Scale; TK: *Taijin-Kyofu-sho*; SAD: social anxiety disorder.

## Data Availability

The data used to support the findings of this study are available from the corresponding author upon request.

## Abbreviations

TK: * Taijin-Kyofu-sho*
SAD: Social anxiety disorder
ADHD: Attention deficit hyperactivity disorder
ASRS: Adult ADHD Self-Report Scale
LSAS-J: Japanese version of Liebowitz Social Anxiety Scale.
